# Mitochondrial Dysfunction in Aging and Diseases of Aging

**DOI:** 10.3390/biology8020048

**Published:** 2019-06-17

**Authors:** Richard H. Haas

**Affiliations:** 1 Department of Neurosciences, University of California San Diego, San Diego, CA 92093-0935, USA; rhaas@ucsd.edu; 2 Department of Pediatrics, University of California San Diego, San Diego, CA 92093-0935, USA

Mitochondria have been increasingly recognized as the important players in the aging process. Most aging-related diseases, particularly, neurodegenerative diseases, have mitochondrial involvement. A PubMed search for mitochondria and aging lists 704 articles in 2018. This is not surprising as mitochondria are involved not only in energy production through oxidative phosphorylation but also play an important role in intracellular homeostasis, calcium balance, and the metabolism and interconversion of our dietary substrates, fats, proteins, and carbohydrates, in the fed and fasting states. They have an important role in signaling their metabolic state to the nucleus and to other cells in response to stress. Mitochondria have their own protein synthetic apparatus and replicate themselves, pathways readily disrupted in disease and aging. They are constantly involved in fusion and fission, the balance of which is essential for cell health. These organelles participate in apoptosis, they make most of the cell’s free radicals, and they are crucially important for innate immunity. Mitochondrial DNA has an estimated 10-fold greater mutation rate than nuclear DNA and less repair capacity, and this plays an important role in aging and cancer. Mitochondria are impacted by environmental factors and toxins, and different mtDNA haplogroups originally adapted to geographically different origins make an important background contribution to disease. As mitochondria play a critical metabolic role in all organ systems, they are particularly impacted by disease and contribute to the aging process itself. The invited review articles in this special supplement cover most of the common diseases of aging. Enthusiasm for this supplement in *Biology* was driven by the opportunity to review the current state of knowledge about the role of mitochondria in the aging process. The international group of contributing authors includes many of the leaders in their fields. 

This special issue starts with the discussion on the role of mitochondria in a number of critical organ systems; starting with the immune system, the cell danger response and healing, skin aging, the role of coenzyme Q and vitamin D, mitochondria and the retina, and drug toxicity in the geriatric population. The focus then moves to specific diseases of aging the role of mitochondria in diabetes, cancer, cardiovascular disease, and neurodegenerative disorders;amyotrophic lateral sclerosis (ALS), multiple sclerosis, Parkinson’s disease, and Alzheimer’s disease. Finally, an important paper focusing on muscle and aging points out the important therapeutic role of exercise.

Increasingly the role of mitochondria in innate immunity has been studied but, as noted by Peter McGuire [1], the importance of mitochondrial dysfunction in aging and immunity is less discussed. He provides an overview of three main effects of aging on this system, inflammation with aging, susceptibility to viral infections, and declining T-cell function. He points out the role of mitochondrial damage associated molecular patterns (mtDAMPs), which when released from mitochondria as a consequence of stress, apoptosis, or necrosis, trigger caspase-1 activation with the release of pro-inflammatory cytokines. He discusses the increased susceptibility of older adults to viral infections, and the role that mitochondria play in innate immune signaling against viruses and the production of protective type I interferons. Finally, he discusses the hypothesis that T-cell dysfunction in aging is due to a decline in mitochondrial function. 

Robert Naviaux [2] discusses a holistic new model of incomplete healing and its role in aging. He explores the role of mitochondria in the healing process and the effects of aging. He points out that healing involves the cell danger response and that metabolic cross-talk between mitochondria and the nucleus, between neighboring and distant cells via signaling metabokines regulates the completeness of healing. He discusses the causes of cellular stress and the role of mitochondria in the cell danger response, pointing out the critical roles of purinergic and sphingolipid signaling pathways. Finally, this paper discusses that cellular arrest in the various phases of the cell danger response leads to chronic inflammatory and pain syndromes, to susceptibility to bacterial and viral infections, to a variety of aging-related diseases as well as autoimmune disorders, and to neurodegenerative diseases.

Isabella Peixoto de Barcelos and Richard H. Haas [3] review, from a translational perspective, the data regarding the association of CoQ_10_ and aging. They discuss the changes in coenzyme Q levels during the aging process and its putative contribution to aging diseases through a wide variety of metabolic roles. CoQ_10_ functions in membranes throughout the cell where antioxidant and signaling roles predominate. They explore the growing evidence that oxidative stress is a major component of cellular senescence, a multifactorial process that involves DNA, protein, and lipid damage and activation of signaling pathways associated with aging. They discuss the state of the evidence that CoQ_10_ supplementation may be helpful for diseases of aging and aging itself.

Roisin Stout and Mark Birch-Machin [4] review the increasing evidence that mitochondrial dysfunction and oxidative stress contribute to skin aging. They discuss the important mitochondrial role and energy production in cell signaling, wound healing, pigmentation, vasculature homeostasis, and hair growth, as well as defense against infection. They explore the free radical theory of aging in the skin and point out that mtDNA deletions are increased in aged UV exposed epidermis. They review the role of calorie restriction on skin models of aging and the role of mitochondria in the pigmentary changes of aging. Finally, they discuss the role of mitochondria in photoaging, effects of pollution, stress-induced skin wrinkle formation, and hair loss and greying. 

Sunil J. Wimalawansa [5] explores the role of vitamin D and its metabolites in the aging process. He discusses newly recognized functions of vitamin D as controllers of systemic inflammation, oxidative stress, and mitochondrial respiratory function. He reviews the role of the active metabolite 1,25(OH)2D as a gene regulator, inhibiting NF-κB expression, which is thought to play a role in many aging-related disorders, including inflammation and cancer. He explores the evidence that the vitamin D modulates mitochondrial function and functions as a powerful antioxidant. He points out that hypovitaminosis D increases the incidence and severity of several age-related common diseases and metabolic disorders that are linked to oxidative stress, including obesity, insulin resistance, type 2 diabetes, hypertension, pregnancy complications, memory disorders, osteoporosis, autoimmune diseases, certain cancers, and systemic inflammatory diseases. Finally, the importance of worldwide vitamin D supplementation is emphasized.

Janis T. Eells [6] reviews the role of mitochondria in one of the body’s most bioenergetic organs, the retina. The eye is exposed to visible light and has extensive antioxidant protective mechanisms. Aging retinal pigment epithelial cells have impaired mitochondrial function with increased reactive oxygen species production. She notes that aging-related mitochondrial dysfunction causes increased oxidative injury, which, coupled with impaired repair mechanisms, results in retinal dysfunction and retinal cell loss, leading to visual impairment. Janis T. Eells discusses the most common cause of age-related blindness in developed countries, that is, age-related macular degeneration. She discusses the relationship between mitochondrial function and complement factor H, mutations of which are a putative risk factor for age-related macular degeneration. Finally, she discusses the role of mitochondrial dysfunction in diabetic retinopathy and glaucoma.

Yvonne Will, Jefry E. Shields and Kendall B. Wallace [7] bring together extensive academic and pharmaceutical research experience to discuss the role of mitochondria in drug toxicity in the elderly. This is an under-explored topic despite its great importance. They note that ‘Drug-induced mitochondrial toxicity has been described for many different drug classes and can lead to liver, muscle, kidney, and central nervous system injury and, in rare cases, to death’. They discuss the progressive loss of mitochondrial function with age with decreased mitophagy as a cellular surveillance system failure. They point out that drug mitochondrial toxicity has often not been identified in pre-clinical studies because young healthy animals are used rather than aged animals with more susceptible mitochondria. For the many drug classes, the particular drugs with most severe mitochondrial toxic effects can be revealed by in vitro systems where cells are forced to use mitochondrial respiration. Polypharmacy in the elderly, coupled with access to over the counter drugs, which inhibit mitochondrial function, compounds the problem of mitochondrial toxicity. A very useful table of commonly used drugs and their mitochondrial effects has been provided. Finally, the important effects of lifestyle and diet on susceptibility to mitochondrial toxicity is discussed.

Magdalene K Montgomery [8] provides a valuable review of the role of mitochondria in obesity and type 2 diabetes. A discussion of the role of mitochondria in diabetic organ damage focuses on diabetic cardiomyopathy. A review of the role of mitochondria in insulin resistance finds that while most of the antidiabetic drugs increase mitochondrial biogenesis with an improvement in insulin efficacy, particularly, those that modulate peroxisome proliferator-activated receptors, there are mitochondrial toxic effects caused by many antidiabetic drugs. Finally, she explores the beneficial or detrimental role of intercellular exchange of mitochondria, mitochondrial DNA, and mitochondrial fragments through the exchange of exosomes and through nanotubes.

Jason Duran, Armando Martinez and Eric Adler [9] discuss cardiovascular manifestations of mitochondrial disease and the role of mitochondria in myocardial ischemia and diabetic cardiomyopathy. Cardiomyocytes are among the most energy dependent cells in the body. They review the mitochondrial changes with aging and the effects on the heart. They then discuss the cardiac and cardiovascular manifestations of canonical mitochondrial syndromes. In these disorders, hypertrophic and dilated cardiomyopathies are commonplace, and a variety of cardiac conduction defects can be life-threatening. A discussion of mitochondrial dysfunction in cardiac ischemia details the role of mitochondrial dysfunction and oxidative stress in reperfusion injury, which increases the size of myocardial infarction through necrosis and mitochondrially mediated apoptosis. Finally, the mitochondrial role in diabetic cardiomyopathy is discussed.

The next article by Nima B. Fakouri, Thomas Lau Hansen, Claus Desler, Sharath Anugula and Lene Juel Rasmussen [10] addresses the interactions between mitochondria and cancer. The authors focus their review on genomic instability, dysregulation of cellular energetics, and mitochondrial function. They note that DNA damage, secondary to oxidative stress produced by reactive oxygen and nitrogen species, activates the DNA damage response (DDR), which is an energy-dependent process. They then elegantly discuss how the DDR can both activate and impair mitochondrial function, the latter through poly (ADP-ribose) polymerase enzyme hyper-activation. They next discuss the role of mitochondria-nuclear signaling in aging and cancer, along with the role of ROS. Mitochondria have an epigenetic role, and, in cancer, mtDNA mutations are associated with a poor prognosis―and they note a correlation between mitochondrial respiration, cytosolic dNTP pools, and chromosomal instability. In summary, the connection between mitochondria and cancer is complex but, as the authors note, ‘the hallmarks of cancer include genomic instability, dysregulation of cellular energetics, and mitochondrial dysfunction, which also are common pathways important for cellular aging’.

Moving on to neurodegenerative diseases, the review, by Veronica Granatiero and Giovanni Manfredi [11], discusses the role of mitochondrial dysfunction in the devastating disorder amyotrophic lateral sclerosis (ALS). They note that neurons are very energy dependent, and mitochondrial transport and turnover is critical for neuronal and axonal health. In both genetic ALS and 90% of sporadic cases, mitochondrial dysfunction is manifest by changes in fusion, fission, and transport. These protein ‘motors’ are ATP dependent. They note that in ALS, microtubular kinesin anterograde mitochondrial transport and dynein retrograde transport are both disrupted. The ATP/ADP ratio is an important component of the signaling system for mitochondrial transport. The hypothesis that protein aggregates in ALS bind to and damage mitochondria causing dysfunction is discussed. Gene mutations linked to ALS are involved in mitochondrial quality control. The authors note that a recent study shows that the actin cytoskeleton plays a part in isolating damaged mitochondria from the rest of the network. Mitophagy is increased, and Parkin levels are decreased, in animal models and human ALS, suggesting that the decline in Parkin protein is related to mitophagy. Whether mitochondrial dysfunction in ALS is a cause or an effect remains unclear, but mitochondrial fusion, fission, and transport have an important role in this disease.

Isabella Peixoto de Barcelos, Regina M. Troxell and Jennifer S. Graves [12] discuss the role of mitochondria in multiple sclerosis (MS). Decreases in oxidative phosphorylation and mitochondrial transport have been documented. MS is an inflammatory disorder with acute and chronic phases, involving both mitochondrial innate immunity and chronic inflammation followed by neurodegeneration. They note that mitochondrial dysfunction occurs as a consequence of inflammation and oxidative stress with ROS production. Nucleic acid, protein, and lipid damage ensue. A fall in ATP production ultimately triggers mitochondrially mediated apoptosis. Mitochondrial stress impairs oligodendrocyte function. Studies in human cortex confirm increased levels of mtDNA mutations, mtDNA depletion, and impairment in complex I and III enzyme activity. Mitochondrial changes in animal models are reviewed. Experimental autoimmune encephalomyelitis, an animal model of MS, shows mitochondrial swelling and dysfunction with some evidence for antioxidant rescue. The authors note that some mtDNA diseases due to point mutations, such as Leber’s Hereditary Optic Neuropathy, can have an MS phenotype, and MS-like demyelinating disease has been reported in a variety of mitochondrial-nuclear gene defects, including PolG and OPA1. Large population studies have failed to confirm an increased incidence of mtDNA mutations in the MS population, although the JT haplogroup does have an increased MS risk. Finally, the authors discuss the role of mitochondrial therapies in MS.

Chun Chen, Doug M. Turnbull and Amy K. Reeve [13] provide a current review of the mitochondrial role in Parkinson’s disease (PD). Evidence for mitochondrial involvement stretches back 40 years. They discuss mitochondrial pathways involved in PD and the rapid growth of knowledge resulting from genomic sequencing of familial cases. Evidence for complex I deficiency in animal models and human disease is discussed. High levels of mtDNA clonal deletions are found in substantia nigra neurons in normal aging and PD, with ROS as a likely contributing factor. This aging-related mtDNA pathology in substantia nigra likely contributes to susceptibility to PD, and alpha-synuclein can increase mitochondrial membrane permeability, ROS production, and cell death. The authors discuss recent data on cytosolic calcium oscillations with the import of calcium into mitochondria, which likely plays a role in cell death. Mutations in two genes *VSP35* and *CHCHD2,* which impair the mitochondrial function, are responsible for autosomal dominant PD. These and the roles of mutations in PINK1 and Parkin in mitophagy are discussed. Finally, we are treated to a detailed discussion of mitochondrial turnover and dynamics and the putative role of protein aggregation in PD. The authors conclude that mitochondrial complex I deficiency plays a key role in PD, and the likely causes are elegantly discussed.

Ian Weidling and Russell H. Swerdlow [14] review the evidence for a mitochondrial role in Alzheimer’s disease (AD). Starting with longstanding evidence of brain hypometabolism, they discuss findings of mitochondrial electron transport enzyme deficiencies and, in particular, cytochrome oxidase (COX) deficiency in Alzheimer’s brain. Recent studies confirm mitochondrial structural abnormalities in Alzheimer’s brain, likely the result of a fusion-fission malfunction, which may be related to amyloid beta accumulation. An interesting reciprocal relationship between mtDNA deletions and aging noted in AD brain is discussed, and the evidence for increased oxidative injury in AD brain compared to age-matched controls is reviewed. Cybrids created from AD mtDNA show similar changes to the brain with COX deficiency. A mitochondrial interaction with Alzheimer’s protein aggregates is discussed. Mitochondrial toxins can induce the formation of AD-like Tau alterations, and, in mouse models, mitochondrial dysfunction precedes amyloid plaque accumulation. The authors note that it is unclear whether mitochondrial changes are the cause or effect of abnormal protein accumulation in AD. Amyloid beta inhibits COX activity, and Tau accumulation disrupts mitochondrial transport. ApoE4 overexpression impairs electron transport complexes. The role of mitochondria in clearing protein aggregates is reviewed. Finally, the multiple effects of mitochondrial dysfunction and the Integrated Stress Response (activated in AD) on gene expression are discussed. The authors conclude that given the multiple lines of evidence of mitochondrial dysfunction in AD—this is a reasonable therapeutic target.

This special edition on mitochondrial dysfunction in aging concludes with a discussion of treatment. Sarcopenia is an inevitable consequence of aging. Mats I Nilsson and Mark A Tarnopolsky [15] detail the role of exercise as a treatment for mitochondrial aging. An overview of mitochondrial evolution leads on to a discussion of the homeostatic role of mitochondria and their role in defense against the three main aging changes, oxidative injury, protein aggregation, and inflammation. An integrated system’s hypothesis of aging is developed with mitochondria playing a central role. The role of mitochondrial ROS is discussed, and the authors point out that oxidative phosphorylation decline is an aging phenomenon in all species along with a variety of structural and functional mitochondrial changes. Age-related protein aggregation and lipofuscin accumulation result in an accumulation of cellular debris impervious to lysosomal and proteasomal degradation. The mitochondrial role in the chronic inflammation of aging is explored. The authors then turn to the role of exercise in correcting the cellular decline of aging. A convincing case is made for the benefit of exercise, slowing aging-related changes, including reducing intracellular danger signals, rejuvenating mitochondria, assisting with intracellular garbage clearance, and decreasing aging-related inflammation. 
